# Deriving anti-epidemic policy from public sentiment: A framework based on text analysis with microblog data

**DOI:** 10.1371/journal.pone.0270953

**Published:** 2022-08-01

**Authors:** Sijia Zhao, Lixuan Chen, Ying Liu, Muran Yu, Han Han

**Affiliations:** 1 School of Economics and Management, Universiity of Chinese Academy of Sciences, Beijing, China; 2 College of Biological Science, University of California, Davis, California, United States of America; 3 Shanghai SAIC Mobility Technology and Service Co. LTD., Shanghai, China; Stanford University, UNITED STATES

## Abstract

Microblog has become the “first scenario” under which the public learn about the epidemic situation and express their opinions. Public sentiment mining based on microblog data can provide a reference for the government’s information disclosure, public sentiment guidance and formulation of epidemic prevention and control policy. In this paper, about 200,000 pieces of text data were collected from Jan. 1 to Feb. 26, 2020 from Sina Weibo, which is the most popular microblog website in China. And a public sentiment analysis framework suitable for Chinese-language scenarios was proposed. In this framework, a sentiment dictionary suitable for Chinese-language scenarios was constructed, and Baidu’s Sentiment Analysis API was used to calculate the public sentiment indexes. Then, an analysis on the correlation between the public sentiment indexes and the COVID-19 case indicators was made. It was discovered that there is a high correlation between public sentiments and incidence trends, in which negative sentiment is of statistical significance for the prediction of epidemic development. To further explore the source of public negative sentiment, the topics of the public negative sentiment on Weibo was analyzed, and 20 topics in five categories were got. It is found that there is a strong linkage between the hot spots of public concern and the epidemic prevention and control policies. If the policies cover the hot spots of public concern in a timely and effective manner, the public negative sentiment will be effectively alleviated. The analytical framework proposed in this paper also applies to the public sentiment analysis and policy making for other major public events.

## Introduction

The novel coronavirus pneumonia (“COVID-19”) has spread rapidly to 31 provinces on the Chinese mainland, Hong Kong, Macao and Taiwan as well as 25 countries around the world since its outbreak, and the World Health Organization (WHO) has declared it a “Public Health Emergency of International Concern (PHEIC).” As an important hot event related to social security, the spread of COVID-19 has changed public life and attracted great attention from the public [1]. During the outbreak of the COVID-19 epidemic, microblog has been used at an unprecedented scale, and it has become an important platform for obtaining and exchanging information [2]. The content about COVID-19 released by the public and on We Media has attracted a large number of reposts, comments and likes, which make microblog the “first scenario” under which the public pay attention to the development of the epidemic and express their opinions and emotions. Therefore, timely perception and collection of public sentiment and its effective analysis through microblog is of important reference value for the government to release information, ease public sentiments, avoid irrational panic-related risks and accurately prevent and control the epidemic [3].

Existing literature has made much progress in perceiving and collecting public sentiments in public health events through microblog data. Golder and Macy [4] published a paper in Science, pointing out that users’ emotional change patterns can be effectively extracted and analyzed from a large number of text messages posted on Twitter. Microblog represented by Twitter serves as an effective tool for studying information related to public health events and can be used to track public sentiments and grasp their development trends [5–7]. Ji et al. [8, 9] believed that it is very important to track the spread trends of the epidemic and identify the hot spots of public concern. They proposed the Epidemic Sentiment Monitoring System (ESMOS), which adopted Tweets data to measure public concerns through two-step sentiment classification. Seltzer et al. [10] found that, among 500 images posted on Instagram, 51% showed fear and negative sentiments about the virus. Alessa et al. [11] believed that social network data can effectively track disease outbreaks, and found that sentiment analysis can improve the accuracy of epidemic-related tweets classification. Peng et al. [12] analysis of wuhan COVID-19 cases in time and space distribution by using sina weibo data, and proposed effective public health prevention strategies for urban spatial optimization. Li et al. [13] used the online ecological identification (OER) method based on machine learning prediction model to sample and analyze weibo posts from 17,865 active weibo users. The study found that after the outbreak was announced, users’ negative emotions and sensitivity to social risks increased, and people were more concerned about family and their own health. Duan et al. [14] used 6.3 million text data extracted from official news media and Sina Weibo to capture covid-19-related sentiment. The study found that COVID-19 sentiment has a positive predictive effect on stock return rate and turnover rate, and margin and short selling activities are actively strengthened with the growth of sentiment. Wang et al. [15] uses machine learning technology to track the expressed emotions at the national and local (state/provincial) levels with high temporal and spatial granularity based on relevant data of Twitter and Weibo. The study found that COVID-19 outbreak caused global express mood fell sharply, followed by asymmetry, slow recovery. These studies prove that microblog is effective in collecting and perceiving public sentiments, and it is feasible to use machine learning and text mining technology to analyze microblog data.

The arrival of the era of big data has brought massive, complex and ever-increasing data [16, 17], and the problem can be better solved by simplifying and effectively analyzing the data. This study aims to analyze the rules of changes in public sentiments during the epidemic, explore the sources of negative sentiment, and provide a reference for the government and official media to release scientific information and channel public sentiments. Firstly, the research framework of data collection, processing, analysis and application was proposed. A total of 200,000 microblog data from Sina Weibo (Weibo), which is the most popular microblog website in China from Jan. 1 to Feb. 26, 2020, and a dictionary suitable for Chinese-language sentiment classification was constructed. The Baidu API was employed to calculate sentiment scores, and the changes in public sentiments along with time were analyzed. Then, an analysis of correlation between public sentiments and epidemic-related indicators was conducted. Finally, the LDA model was used to extract different topics of public sentiments at different stages, identify the sources of negative sentiment, and provide a reference for the government to guide public sentiments.

## Methods

**Fig 1:**
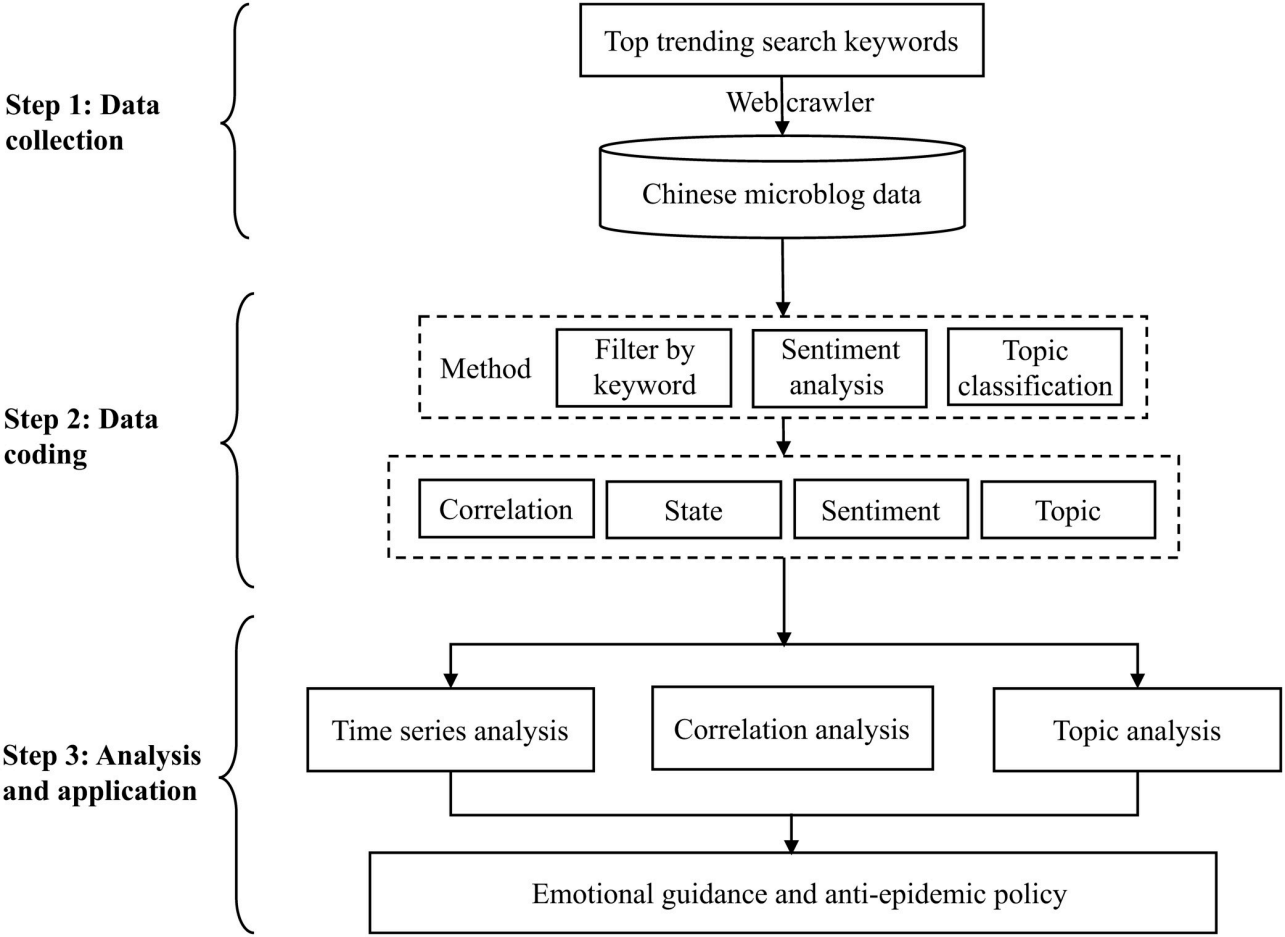
The framework of Weibo public sentiment analysis.

Based on microblog data, this paper mines social network information, identifies and analyzes public sentiments. The analysis process is mainly divided into three steps: data collection, data processing, as well as data analysis and application. The analysis method framework is shown in Fig 1.

### Data collection

Sina Weibo (Weibo) is the most popular microblog website and the largest portal in China [18]. As the mainstream social networking platform in China, Weibo registered 516 million monthly active users as of the end of 2019, with a high degree of data openness as well as rich ideas and opinions. Since the outbreak of COVID-19 in early 2020, a large number of news, posts and discussions related to the epidemic have been published on Weibo, making it the representative data source for analyzing public sentiments during the epidemic. We used the username and password to access to Sina Weibo by imitation login, and used web crawler technology to get data. a total of about 200,000 Weibo posts from Jan. 1 to Feb. 26 were collected in this paper. The data collection process consists of the following steps:

First, three key words, namely “novel coronavirus”, “epidemic disease” and “pneumonia” were selected according to the hot search and Weibo’s recommendation function during the epidemic. Then, the data indicators and formats were determined to be collected. Weibo’s advanced search function was used to obtain the relevant Weibo information found through each key word, including date of release, publishers, content of release, the number of comments and the number of likes. Finally, a web crawler was designed, and the above three key words were adopted as search words to automatically get all the index data of Weibo corresponding to each key word in the specified timestamp, which was saved as time series data.

The diagnostic data of COVID-19 in China used in this paper is sourced from the website of the National Health Commission (NHC) of the People’s Republic of China (http://www.nhc.gov.cn/).

### Data coding

As Weibo data is non-structured, the Weibo data collected in this paper was coded, which is the basic task of data analysis. Weibo text was coded from four aspects: correlation, state, sentiment and topic. Each Weibo post was marked with a code consisting of four dimensions (r, s, o, t).

Correlation(r): Determine whether the Weibo text is related to COVID-19. As the collected data contains advertising information with epidemic keywords, it is necessary to determine whether Weibo text is related to COVID-19. If yes, the value is 1; if not, it is 0. According to the common advertising keywords, such as “after discount”, “price”, “¥”, “order”, etc., we establish a database of noise words. If Weibo contains any noise word, the words will be considered irrelevant to “COVID-19” and thus deleted from the original data.

State(s): Judge the status of Weibo text. Weibo text is mainly divided into two categories: news posts, which refer to Weibo posts issued by authorities, institutions and media, while non-news posts refer to those published by the public (individual users) on Weibo to express their feelings or disclose their experiences. The status dimensions of Weibo are identified by authorities through certified tags and news-specific key words.

Sentiment(o): Judge the sentiment tendency of Weibo text, which is divided into three levels: positive, negative and neutral. Recognition method: identify the emotions of 4,000 Weibo posts, including 2,000 pieces of posts with positive sentiment and 2,000 pieces of posts with negative sentiment. A total of 4,000 posts were selected as the training set and Baidu NLP was used for training. The process of emotion recognition will be described in detail below.

Topic(t): Determine the topics of Weibo. The topics related to COVID-19 include: epidemic development, epidemic prevention information and measures, disease and help information. These topics are identified and marked through word segmentation, keyword extraction and clustering analysis.

### Sentiment analysis

With comparatively high representativeness, the sentiment tendency of personal Weibo posts can expose the fluctuations of public sentiments during the epidemic to a certain extent. In this paper, the sentiment tendency analysis of collected epidemic-related Weibo text data is mainly divided into two steps: the construction of sentiment dictionary and the determination of sentiment polarity of the text.

#### Sentiment dictionary

Lexical features are the key to many methods of sentiment analysis and opinion exploration [19]. Sentiment elements of Weibo text data usually include sentiment words with tendencies, degree adverbs and some negative words that can change the tendencies. The construction of a sentiment dictionary is not only to store the above complex textual sentiment elements in the form of vocabulary, but also to identify them as the basis for judging sentiment polarity [20].

First, the Weibo text data related to the epidemic was cleaned, and punctuation marks and noise words such as “forwarding” and “http.” were deleted. Then, a total of 4,000 pieces of Weibo text data were extracted from the total samples and marked by hand, including 2,000 pieces of positive sentiment and 2,000 pieces of negative sentiment, which form the final sentiment dictionary.

#### Sentiment analysis

In this paper, the Baidu Sentiment Analysis API is selected to calculate the sentiment value of Weibo text. This tool uses the Bi-LSTM sentiment classification model to judge sentiment tendencies based on semantics, thus avoiding the limitations of traditional feature-based engineering classification. The calculation result ranges from 0 to 1. When the text score is around 0.5, the text sentiment will be neutral. When the result is greater than 0.5, the text sentiment will be more positive, and the larger the result value is, the more positive the text will become. When the result is less than 0.5, the text sentiment will tend to be negative; and the closer it is to 0, the more negative the text sentiment will be.

In this paper, the above methods are used to identify the sentiment polarity of Weibo text. A total of 4,000 manually-marked terms and expressions in the sentiment dictionary are divided into training sets and test sets according to the ratio of 7:3, and uploaded to the Baidu open platform for model training, detecting recognition accuracy and adjusting parameters. After training, the recognition accuracy of the model for text sentiment polarity reaches 85%, and then the model is used to judge the sentiment polarity of all Weibo text data.

### Topic classification

Through the Latent Dirichlet Allocation (LDA) model, a Weibo topic classification model was constructed in this paper, and the topics of Weibo text data related to the COVID-19 epidemic were extracted and classified. The extraction and classification of Weibo topics are divided into two steps: text segmentation and topic induction & classification.

#### Text segmentation

As there is no obvious spacing between words in Chinese texts, it is necessary to segment Chinese texts before topic classification. Jieba, a Python toolkit for Chinese text segmentation, is used to segment Weibo text data based on Harbin Institute of Technology’s Common Words Dictionary, and the Baidu Input Method Editor, Sogou Input Method, Sogou Common Words, Tencent Input Method and other dictionaries are used as basis. On the one hand, the use of multiple dictionaries can deal with some new online vocabulary; on the other hand, it can prevent the negative words with sentimental change tendency from being separated; for example, “unhappy” is separated into “not” and “happy.”

#### Topic classification

LDA is an unsupervised machine learning technology, which can be used to identify the hidden topic information in large-scale document collection or corpus. Each document in the LDA model represents a probability distribution composed of some topics, and each topic represents a probability distribution composed of many words [21]. For each document in the corpus, LDA defines the following generation process:

1. For each document, extract a topic from the topic distribution;
2. Extract a word from the word distribution corresponding to the drawn topic;
3. Repeat the above process until each word in the document is traversed.

The core formula of LDA is as follows:

$$\begin{array}{c} P(w|d)=P(w|t)\times P(t|d), \end{array}$$ (1)

Perform a *P*(*w*|*d*) calculation of all words *w* in all documents *d* in the document set *D* and re-select the topic *t* as an iteration. After iterations for n times, we will converge to the results required by LDA.

In this paper, we extract key words from epidemic-related Weibo text and use LDA topic model for training and analysis; we have extracted 20 topics in an unsupervised way, and each topic stores 20 key words; we use the extracted key words to match the original text of Weibo, merge and name the topics with similar contents, and make statistics on the time and frequency of each topic.

## Results

### Time series analysis

**Fig 2:**
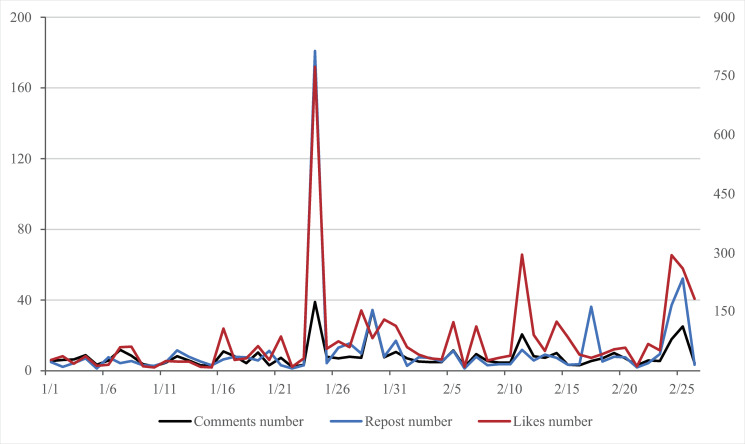
Time series analysis of Weibo discussion heat.

**Fig 3:**
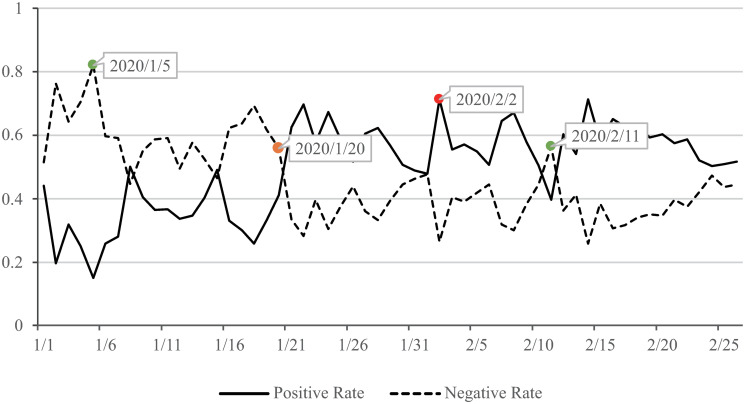
Time series analysis of personal Weibo.

This section combines Weibo data with the results of sentiment analysis of Weibo text for descriptive statistical analysis and time series analysis. The average number of comments and likes per Weibo post every day (Fig 2) and the ratio of positive sentiment to negative sentiment on Weibo every day were calculated (Fig 3).

The number of comments, likes and reposts on Weibo can reflect the popularity of public discussion on the epidemic situation [22, 23]. It can be seen from the data in Fig 2 that during the period from Jan. 1 to Feb. 26, 2020, the overall trend of the average number of comments, likes and reports per Weibo post per day is relatively consistent. In a selected period of time, the number of comments peaked four times,the number of likes peaked three times and that of reports three times, both of which peaked on Jan.24, the New Year’s Eve. The peaks of comments on Jan.24 and 29 were quite close to each other, but there was no peak of likes and reports on Jan.29. Through the comparative analysis of word frequency, it was found that there is little difference in the contents of Weibo in the two days, which mainly include “pay tribute to the front-line medical staff” and “wish you a safe and healthy New Year.” This suggests that after days of content homogenization, the level of public empathy faded faster than the voice of discussion. The other two peaks in the number of comments and likes occurred on Feb.11 and 25 respectively, which were the second and third 14-day control cycles for the COVID-19 epidemic. According to the word frequency analysis, the topics of “returning to work” and “overseas epidemic situation” aroused heated discussions on Weibo in the two days. Another peak in the number of reports was on Feb.17, when the topic “Aid to Wuhan” received a large number of reports.

Fig 3 shows the fluctuation trend of public sentiments in the selected period of time. Generally speaking, the proportion of positive sentiment is higher than that of negative sentiment. The general attitude of the public towards “novel coronavirus pneumonia” shows a positive trend. Negative sentiment peaked on Jan.5, while positive sentiment peaked on Feb.2.Through word frequency analysis, it can be found that on Jan.5, when the epidemic was in the initial stage, the discussion on Weibo mostly involved “eliminate SARS,” “trace back,” “virus” and other similar contents. This high degree of uncertainty triggered negative sentiment among the public. Till Feb.2, the government had implemented prevention and control measures for nearly two weeks, and the public’s understanding of the epidemic situation had deepened. Most contents of Weibo posts contained positive words such as “fight,” “prevention and control” and “firmly believe,” and the positive sentiment reached the peak. As we can see intuitively from Fig 3, Jan.20 is a key turning point. Before Jan. 20, personal Weibo posts related to the epidemic were mainly of negative sentiment, while after Jan.21, these Weibo posts mainly contained positive sentiment. According to the news of the day, on Jan.20, Zhong Nanshan, an expert in respiratory medicine of the National Health Commission and head of the high-level expert group, confirmed for the first time that the novel coronavirus infected by novel coronavirus carriers can be transmitted from person to person. At the same time, strict prevention and control measures were implemented nationwide. This shows that the government’s information transparency and active prevention and control measures during the epidemic can effectively reduce the negative sentiment of the public. It is worth noting that on Feb. 11, the proportion of negative sentiment among the public was relatively high, which was the only occurrence after Jan. 20 when it exceeded positive sentiment. That day, there occurred many family cases in Beijing and suspected airborne cases in Hong Kong, showing that some negative news during the prevention and control period would still cause negative sentiment among the public.

### Correlation analysis

To verify the correlation between public sentiments and the development of the COVID-19 epidemic, this paper uses Granger causality test to analyze the statistical correlation between different Weibo indicators and COVID-19 epidemic indicators.

Here, the proportion of official Weibo text and that of personal negative sentiment in Weibo text are selected as Weibo indicators.

*O* represents the proportion of official Weibo text.

*N* represents the proportion of personal negative sentiment related to COVID-19 in Weibo text.

The growth rate, mortality rate and cure rate are used to reflect the COVID-19 epidemic situation. In order to show the growth rate of confirmed cases, one indicator, namely, the number of newly confirmed cases per day divided by the number of existing confirmed cases that day, was constructed. Considering the stability of data, the growth rate, mortality rate and cure rate are adjusted by first-order difference, and the following three indicators are obtained.

*R*_*growth*_ is an incremental indicator for the growth rate of confirmed COVID-19 cases.

*R*_*death*_ is an incremental indicator for the mortality rate of COVID-19 patients.

*R*_*cure*_ is an incremental indicator of the cure rate of COVID-19 patients.

In this paper, the optimum lag stage of the model is determined by referring to AIC and BIC criteria. The results are shown in Table 1.

**Table 1:** Granger test result of different Weibo indicators and COVID-19 epidemic indicators.

Null Hypothesis	F-test	P-value
The official Weibo text *O* does not Granger-cause the growth rate *R*_*growth*_	0.09	0.7643
The growth rate *R*_*growth*_ does not Granger-cause the official Weibo text *O*	1.48	0.2414
The official Weibo text *O* does not Granger-cause the mortality rate *R*_*death*_	0.48	0.6976
The mortality rate *R*_*death*_ does not Granger-cause the official Weibo text *O*	11.31	0.0018*
The official Weibo text *O* does not Granger-cause the cure rate *R*_*cure*_	6.63	0.0143*
The cure rate *R*_*cure*_ does not Granger-cause the official Weibo text *O*	4.98	0.0129*
The negative sentiment *N* does not Granger-cause the growth rate *R*_*growth*_	4.98	0.0129*
The growth rate *R*_*growth*_ does not Granger-cause the negative sentiment *N*	0.76	0.3884
The negative sentiment *N* does not Granger-cause the mortality rate *R*_*death*_	3.59	0.0250*
The mortality rate *R*_*death*_ does not Granger-cause the negative sentiment *N*	1.47	0.2337
The negative sentiment *N* does not Granger-cause the cure rate *R*_*cure*_	0.76	0.3880
The cure rate *R*_*cure*_ does not Granger-cause the negative sentiment *N*	0.34	0.5626

Note:

*p < 0.05.

According to the test results (Table 1), we can find that there are different Granger causality relationships between the proportion of official Weibo text and that of personal negative sentiment in the Weibo text and the COVID-19 epidemic index. At the 5% significance level, the proportion of official Weibo text show that the proportion of official Weibo text has nothing to do with the increase of confirmed cases, while the results of Granger causality of mortality indicate that official Weibo text is just a kind of notification and response to the development of the epidemic situation. Cure rate is also the Granger reason for the official Weibo, this shows that the cure rate can cause the change of the official Weibo in statistical sense. This can also be interpreted in reality, the official media hope to post the good news of the cure rate increase to the public, to ease the public’s fear for the COVID-19, so the higher cure rate, the official Weibo will be higher. The proportion of personal negative sentiment in the Weibo text forms the Granger reason for the increase of confirmed cases and mortality; otherwise, it would be insignificant. This shows that the negative sentiment of the public can cause the change in the growth rate of confirmed cases and mortality rate in the COVID-19 epidemic in a statistical sense. However, there is no statistical correlation between the proportion of personal negative sentiment in Weibo text and the cure rate.

### Topic analysis

In order to further understand the sources of public negative sentiment during the epidemic, we identified and classified the topics of personal negative sentiment in the Weibo text, and divided the Weibo contents of personal negative sentiment into five topics: “emotional performance,” “daily life issue,” “epidemic information,” “supply issue” and “treatment information,” and 20 sub-topics such as “afraid and fruitful” and “disappointed.” In Table 2, a statistical analysis is made on each topic, while in Table 3, examples are given for the key words and Weibo contents of each topic. Statistics show that “emotional performance” accounts for the highest proportion of negative sentiment, namely 25.8%, followed by “daily life issue,” accounting for 25.02%, both of which are very similar. Among them, “emotional performance,” “daily life issue” and “epidemic information” account for more than 70%, indicating that the negative sentiment of the public during the epidemic mainly came from the expression of emotions, the impact of the epidemic on daily life and related epidemic information.

**Table 2:** Statistics of Weibo on different topics.

Topic	Emotional performance	Daily life issue	Epidemic information	Supply issue	Treatment information
Number	2,663	2,583	2,472	1,807	797
Percent	25.80%	25.02%	23.95%	17.51%	7.72%

**Table 3:** The topics, key words and examples of negative sentiment.

Topic	Subtopic	Key words	Examples
Emotional Performance	1.Afraid and fearful 2.Disappointed 3.Not enough attention	afraid, panic, boring, disappointed, visiting relatives, earnestly	1.Let me home after the exam. I’m afraid of Wuhan. What’s pneumonia? 2.After the outbreak, I must go out with your friends for dinner, shopping and disco dancing. Life as a pig is too boring.
Daily Life Issue	1.Get back to normal life 2.Work resumption 3.Dare not go out 4.Don’t eat bushmeat	finish fastly, want to go out, return to work, the prevention and control, masks, crowded, respect for nature	1.There has never been a better time to wish the epidemic over. I really want to get back to my normal life. 2.I want to go home!! Wuhan has pneumonia, I am a little afraid, go out also have to wear a mask.
Epidemic Information	1.The lockdown of Wuhan 2.Outbreak in Japan 3.Spreading rumors 4.Too late to publish 5.Inflection point 6.Person-to-persontransmission 7.The cause of the infection is unknown	the lockdown of city, isolation, escape, Japan, the Olympic Games, rumors, ignorance, bureaucracy, right to know, panic	1.I don’t know why it is not good to stay in Wuhan, also driving tour. There are eleven people from Wuhan in my town. Okay, now we need to isolate and disinfect. 2.I can’t believe that. I wanted to go to Japan after the outbreak. Now, I’m afraid it’s just the beginning. I hope it won’t affect the Olympic Games.
Supply Issue	1.Shortage of medical supplies 2.Masks are not available 3.To snap up	masks, medical personnel, not enough, out of stock, price gouging, Shuang Huang Lian	1.The whole of Hubei is about to be lockdown! Can the logistics be guaranteed? Are the supplies in place? Are you protected? Medical workers are in a difficult position. The situation in Wuhan and Hubei is so bad. 2.Medical surgical masks have not been available since the outbreak, let alone the N95. My stock of medical nursing masks was snapped up online a few days before the lockdown of the city. After I paid for them, they were sold out.
Treatment Information	1.Traditional Chinese Medicine (TCM) 2.Mental disorder 3.Patients for help	Traditional Chinese medicine(TCM), serious, mental disorders, patients, help, death	1.Is the person who blackens Chinese medicine a fool? Or not Chinese! 2.After watching the super topic of pneumonia patients, I have no other feelings except helplessness and heartache. There is no one to save them, they are struggling and struggling. Hopefully the sick can get help.

**Fig 4:**
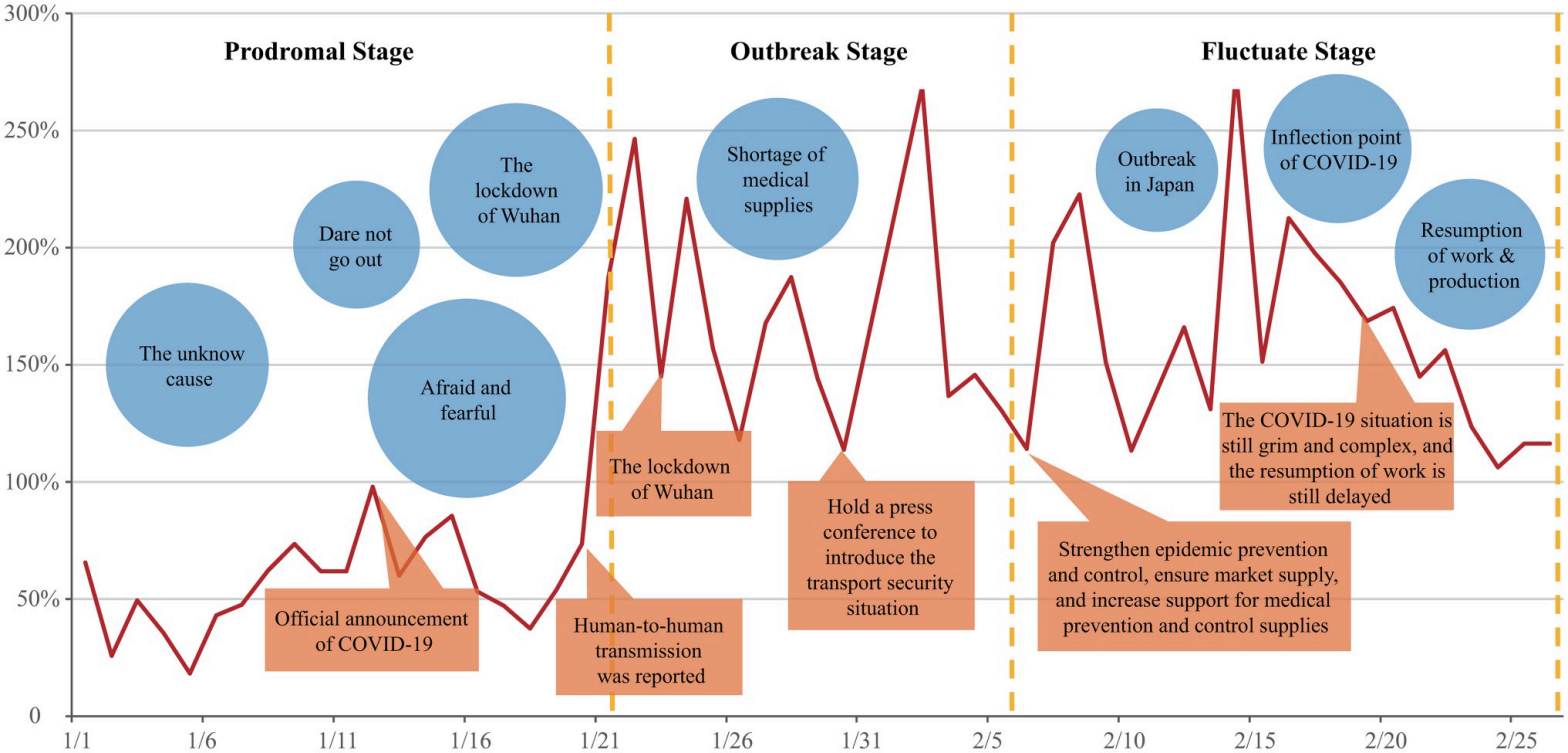
Topic analysis of public sentiments during COVID-19 epidemic.

According to the crisis life cycle theory proposed by the American crisis management scientist Steven Fink, sudden public health crisis usually follows a specific four-stage life cycle model: potential stage, outbreak stage, fluctuate stage and recovery stage. Ahmed and Opoku’s [24] research on technology-supported learning and teaching in crisis period takes the life cycle theory of emergency management as the research perspective and divides the cycle into four parts: mitigation, preparation, response and recovery. Combined with the development of the COVID-19 epidemic in China, this paper divides the sample period into three stages: “prodromal stage,” “outbreak stage” and “fluctuate stage” (Fig 4). In order to better reflect the impact of government information on public sentiments, the public sentiment change index was constructed and the topics of public sentiments at each stage were analyzed.

$$\begin{array}{c} CPMI=\frac{ratio_{Positive}}{ratio_{Negative}}\times 100\%, \end{array}$$ (2)

*CPMI* represents the public sentiment change index, *ratio*_*Positive*_ represents the proportion of positive sentiment, and *ratio*_*Negative*_ represents the proportion of negative sentiment.

As shown in Fig 4, it can be found that the public sentiments become better on the whole from Jan. 1 to Feb. 26. In the prodromal stage of the epidemic, the negative sentiment was obvious. When the government changed the name of “viral pneumonia of unknown origin” to “pneumonia infected by novel coronavirus” for the first time on Jan.12, the negative sentiment of the public increased obviously, and Weibo users discussed the topics of “afraid and fearful” most. On the whole, the unknown epidemic situation at this stage caused more negative sentiment, and the public expressed fear and horror for viruses with unknown sources and modes of transmission. This was mainly reflected in the fear of going out and the accusation of spreading rumors and eating bush meat. On Jan. 20, the National Health Commission officially reported the existence of human-to-human transmission, which greatly reduced the public’s worries about the unknown and greatly reduced the proportion of negative sentiment.

After Jan. 22, the epidemic entered the outbreak stage, when the information about the virus was clear, and the negative sentiment of the public began to shift to the epidemic information. The negative sentiment at this stage were mainly aimed at “the lockdown of Wuhan” and “shortage of medical supplies.” After the government officially announced the closure of Wuhan on Jan. 23, the public’s negative sentiment rose obviously. On the one hand, they were worried that the medical resources after the closure would be difficult to access; and on the other hand, they expressed dissatisfaction and anger at those who were still fleeing Wuhan. On Jan.30, after the Joint Prevention and Control Mechanism of the State Council introduced the transport guarantee policies in epidemic prevention and control, the public’s worries about materials were mitigated, and the public’s positive sentiment increased significantly.

From Feb. 6, the epidemic entered the fluctuate stage. On the same day, the Leading Group of the CPC Central Committee for Novel Coronavirus Prevention and Control announced at its meeting that it was necessary to further improve the prevention and control of the epidemic and ensure market supply. Upon the increase of supports for medical prevention and control materials, the proportion of negative sentiment obviously decreased, and the public’s discussion on the inflection point of the epidemic and the resumption of work & production increased. However, after the government announced on Feb. 19 that “the current epidemic situation is still grim and the resumption of work may be delayed,” the proportion of negative sentiment of the public increased, showing that the public was looking forward to resuming work and normal life as soon as possible. Meanwhile, according to the analysis of public sentiments, the epidemic situation in China was relatively stable at this stage, while the epidemic in other countries represented by Japan begun to show up initially.

## Discussion

This study proposes a public sentiment analysis method based on unstructured Weibo data, and applies and analyzes related Weibo contents during the COVID-19 epidemic. This method processes unstructured Weibo data through data coding; deeply mines emotional elements and content elements in text data, analyzes sentiment polarity, identifies and classifies topics on Weibo. This method has proved effective [25–27]. In this paper, the Weibo sentiment dictionary has been constructed by manual tagging, and the accuracy of the Weibo sentiment polarity analysis reaches 85%. Topic analysis can be used to quantitatively analyze emotional sources and has been applied in some previous studies on sentiment analysis of social networks [28, 29]. Hierarchical topic identification was conducted on Weibo text through the LDA topic induction algorithm. A total of five first-level topics and 20 second-level topics were summarized. The results show that this method can effectively identify and analyze the public sentiments of Weibo.

The result of time series analysis shows that the changes in comments and likes are consistent with the development stage of the epidemic situation. The period from Jan.1 to 22 is the initial period of the epidemic, and the discussions on Weibo related to the epidemic were stable with some fluctuations. When the epidemic entered the outbreak stage (Jan. 23 to Feb.5), the discussion popularity on Weibo also increased, and the peaks of comments and likes appeared on Jan.24, the second day after the start of the outbreak stage. In the third stage of the epidemic (Feb. 6 to 26), the newly confirmed cases gradually stabilized and showed a downward trend, and the hot topics of discussions on Weibo were shifted to “return to work,” followed by two discussion peaks again. Compared with the outbreak stage, the discussion peak at the fluctuation stage was lower.

The result of correlation analysis finds that the proportion of personal negative sentiment on Weibo at the confidence level of 5% forms the Granger causality of epidemic indicators, which shows that the fluctuation of public negative sentiment precedes epidemic indicators, and negative sentiment can be used as explanatory variables to predict epidemic indicators. By monitoring the negative sentiment of the public, we can predict the changes of epidemic situation in advance and provide data support for the government to formulate epidemic prevention strategies.

The result of topic analysis shows that the expressions of fear and panic formed the main parts of public negative sentiment during the epidemic. With the development of the epidemic, the sources of negative sentiment of the public changed. The topics of emotional performance were mainly distributed in the initial and outbreak stages, which were more unknown and more likely to cause public panic. The four topic of Daily Life Issue appeared in the early stage and fluctuate stage of the epidemic respectively, indicating that the public would pay more attention to the impact of the epidemic on daily life before and after the outbreak. The proportion of negative sentiment peaked around Jan. 18, and such topics as “Dare not go out” and “Don’t eat bush meat” also appeared on Weibo in the same period, indicating that these two topics caused the most negative sentiment. On Jan.23, the Wuhan Municipal Headquarters for the COVID-19 Prevention and Control released the news that Wuhan was locked down. That day, the posts about “the lockdown of Wuhan” went viral on Weibo and became the topic hotly discussed, only second to “Afraid and Fearful.” However, the proportion of public negative sentiment on Jan. 23 was at a low level, indicating that although the prevention and control measures of the city closure triggered the negative sentiment of some Weibo users, they could generally increase the public’s sense of security and alleviate the public panic during the epidemic. This shows that effective prevention and control measures can ease public panics, and social platforms such as Weibo can be used as effective channels to help the public vent their negative sentiment.

Based on the above analysis results and discussions, the following suggestions on public sentiment guidance strategies can be made:

1. The lack of accurate information will cause public panic, so it is necessary to make use of the authority of media to report on epidemics in a timely and accurate manner. Based on the public demand from Weibo data, we can convey a positive attitude and build a trust mechanism between the media and the public.
2. Public negative sentiment based on Weibo text is helpful to predict the development of epidemics. The government can work out epidemic prevention and control plans by monitoring the negative sentiment of the public so as to ensure that the development of epidemic indicators is within the controllable range.
3. Topic analysis reflects the main sources of public negative sentiment. Based on this result, the government can fully understand the public needs and solve related problems in a specific manner, which is helpful to alleviate the public negative sentiment during the epidemic.

## Conclusion

The concerns, suggestions and emotional tendencies of the public are mined in a timely manner from the massive text data, by which a reference is provided to the government to formulate and adjust public policies, which is of some significance.

Firstly, the sentiment analysis method for Weibo text data proposed in this paper can effectively mine public sentiments from Weibo text data. Combined with sentiment analysis, correlation analysis and topic analysis, the fluctuation trend of public sentiments and the main sources of negative sentiment are extracted from massive Weibo data so as to obtain a more comprehensive understanding of public sentiments during the epidemic.

Secondly, this study provides a reference for the government in its collection and mastery of public sentiments in a timely manner. By analyzing microblog data such as Weibo, the government can better understand the public’s sentiments about COVID-19 epidemic events, discover sensitive information, grasp the hot spots of public concern and the trend of public sentiments, take timely and effective measures to provide emotional counseling, and speed up the epidemic prevention and control.

Thirdly, the sentiment mining and analysis method based on Weibo message data proposed in this paper also applies to the public sentiment analysis of other similar public health events, major catastrophic events and mass social events, so as to provide a data-driven public sentiment counseling strategy support for the government.

In the future research, we will explore multi-source data and further improve research methods; meanwhile, on the basis of current data, we will dig deep into the spatial information in social networks, enrich data contents, and provide more comprehensive and effective sentiment analysis methods and strategic supports.
